# Supplementary material for: ProteinShader: illustrative rendering of macromolecules
Source: BMC Struct Biol. 2009 Mar 30;9:19. doi: 10.1186/1472-6807-9-19 (PMC2672931; doi:10.1186/1472-6807-9-19)
Supplement: Additional file 1 — ProteinShader program without source code. This compressed file contains the complete ProteinShader program including associated libraries, but no source code. A README.txt file gives an overview of the ProteinShader distribution, and the index.html file in the help subdirectory has directions on getting started with the program as well as a set of tutorials. [file 1472-6807-9-19-S1.zip › ProteinShader-beta-0_9_4-binary/help/api/org/proteinshader/structure/Chain.html]

Chain (ProteinShader API)


|  |  |  |
| --- | --- | --- |
| **Overview** \| **Package** \| **Class** \| **Use** \| **Tree** \| **Deprecated** \| **Index** \| **Help** | |  |
| **PREV CLASS**   **NEXT CLASS** | **FRAMES**    **NO FRAMES**     **All Classes** |  |
| SUMMARY: NESTED \| FIELD \| CONSTR \| METHOD | DETAIL: FIELD \| CONSTR \| METHOD |  |


---


## org.proteinshader.structure Class Chain

```
java.lang.Object
  org.proteinshader.structure.Chain
```

**All Implemented Interfaces:**: IDTest, Visitable

---

``` public class Chain extends Object implements IDTest, Visitable ```

A Chain serves as a container for Residues (AminoAcid, Heterogen, and
Water) and Regions (Loop, Helix, and BetaStrand).

---

**Constructor Summary**

`Chain(String chainID,
String modelID,
String structureID)`
  
          Constructs a Chain with the requested chainID, modelID, and
structureID.


| **Method Summary** | |
| --- | --- |
| `void` | `accept(Visitor visitor)`             Accepts a Visitor and does a callback. |
| `AminoAcid` | `addNewAminoAcid(AminoAcidEnum type, String residueID)`             Creates a new AminoAcid with the type and Residue ID given as arguments, adds the AminoAcid to the Chain's collection of AminoAcids, and returns a reference to the new AminoAcid. |
| `BetaStrand` | `addNewBetaStrand(String betaStrandID, String betaStrandNumber, String sheetID, String startResidueID, String endResidueID, int sense, int strandsInSheet)`             Creates a new BetaStrand with the BetaStrand ID given as an argument, adds the BetaStrand to the Chain's collection of BetaStrands, and returns a reference to the new BetaStrand. |
| `Helix` | `addNewHelix(String helixID, String serialNo, String startResidueID, String endResidueID, HelixEnum type)`             Creates a new Helix with the Helix ID given as an argument, adds the Helix to the Chain's collection of Helices, and returns a reference to the new Helix. |
| `Heterogen` | `addNewHeterogen(String name, String residueID)`             Creates a new Heterogen with the name and Residue ID given as arguments, adds the Heterogen to the Chain's collection of Heterogens, and returns a reference to the new Heterogen. |
| `Loop` | `addNewLoop(String loopID, String startResidueID, String endResidueID)`             Creates a new Loop with the Loop ID given as an argument, adds the Loop to the Chain's collection of Loops, and returns a reference to the new Loop. |
| `Water` | `addNewWater(String residueID)`             Creates a new Water with the Residue ID given as an argument, adds the Water to the Chain's collection of Waters, and returns a reference to the new Water. |
| `AminoAcid` | `getAminoAcid(String residueID)`             Returns the AminoAcid with the residueID given as an argument. |
| `BetaStrand` | `getBetaStrand(String betaStrandID)`             Returns the BetaStrand with the betaStrandID given as an argument. |
| `String` | `getChainID()`             Returns the chainID, which is usually a single letter. |
| `Helix` | `getHelix(String helixID)`             Returns the Helix with the helixID given as an argument. |
| `Heterogen` | `getHeterogen(String residueID)`             Returns the Heterogen with the residueID given as an argument. |
| `String` | `getModelID()`             Returns the modelID of the Model this Chain belongs to. |
| `Residue` | `getResidue(String residueID)`             Returns the Residue with the residueID given as an argument. |
| `String` | `getStructureID()`             Returns the structureID of the Structure this Chain belongs to. |
| `Water` | `getWater(String residueID)`             Returns the Water with the residueID given as an argument. |
| `Iterator<AminoAcid>` | `iteratorAminoAcids()`             Returns an Iterator for the AminoAcids held by this Chain. |
| `Iterator<BetaStrand>` | `iteratorBetaStrands()`             Returns an Iterator for the BetaStrands held by this Chain. |
| `Iterator<Helix>` | `iteratorHelices()`             Returns an Iterator for the Helices held by this Chain. |
| `Iterator<Heterogen>` | `iteratorHeterogens()`             Returns an Iterator for the Heterogens held by this Chain. |
| `Iterator<Loop>` | `iteratorLoops()`             Returns an Iterator for the Loops held by this Chain. |
| `Iterator<Water>` | `iteratorWaters()`             Returns an Iterator for the Water molecules held by this Chain. |
| `int` | `numberOfAminoAcids()`             Returns the number of AminoAcids held by this Chain. |
| `int` | `numberOfBetaStrands()`             Returns the number of BetaStrands held by this Chain. |
| `int` | `numberOfHelices()`             Returns the number of Helices held by this Chain. |
| `int` | `numberOfHeterogens()`             Returns the number of Heterogens held by this Chain. |
| `int` | `numberOfLoops()`             Returns the number of Loops held by this Chain. |
| `int` | `numberOfWaters()`             Returns the number of Waters held by this Chain. |
| `String` | `processID(String id, String typeOfID)`             Returns the ID after trimming any leading or trailing whitespace. |
| `void` | `sortBetaStrands()`             After all BetaStrand objects have been added to the Chain, this method can be called to sort the linked hash map (the linked list part does have order) according to the starting residue for each beta-strand. |
| `String` | `toString()`             Returns the chainID, which is usually a single letter. |

**Methods inherited from class java.lang.Object**

`clone, equals, finalize, getClass, hashCode, notify, notifyAll, wait, wait, wait`

**Constructor Detail**

### Chain

```
public Chain(String chainID,
             String modelID,
             String structureID)
      throws InvalidIDException
```

:   Constructs a Chain with the requested chainID, modelID, and
    structureID.
    The chain identifier for a PDB entry is a single character. It
    is usually a capitol letter (A, B, C, *etc.*), but other
    characters could be used. If a structure has only a single chain,
    a PDB file will normally have a blank space as the chain
    identifier, so it would be a good idea for a parser to assign
    some default chain character such as the digit 1. An
    InvalidIDException will be thrown if atomID is null or an empty
    String (leading or trailing white space is trimmed from the
    chainID).

    **Parameters:**: `chainID` - the Chain letter.: `modelID` - the Model number.: `structureID` - the ID code assigned by the Protein Data Bank. **Throws:**: `InvalidIDException` - if chainID is null or does not have at least one non-whitespace character.


**Method Detail**

### accept

```
public void accept(Visitor visitor)
            throws VisitorException
```

:   Accepts a Visitor and does a callback.

    :   **Specified by:**: `accept` in interface `Visitable`
    :   **Parameters:**: `visitor` - the Visitor to do a callback with. **Throws:**: `VisitorException` - if an error occurs while an object is being visited.

---


### addNewAminoAcid

```
public AminoAcid addNewAminoAcid(AminoAcidEnum type,
                                 String residueID)
                          throws MissingAATypeException,
                                 InvalidIDException
```

:   Creates a new AminoAcid with the type and Residue ID given as
    arguments, adds the AminoAcid to the Chain's collection of
    AminoAcids, and returns a reference to the new AminoAcid.
    The AminoAcid will be stamped with the chainID, modelID, and
    structureID of this Chain. The type argument cannot be null,
    or a MissingAATypeException will be thrown. Any leading or
    trailing whitespace will be trimmed off the residueID.

    :   **Parameters:**: `type` - the AminoAcid type as an AminoAcidEnum.: `residueID` - the ID of the AminoAcid. **Returns:**: The newly created AminoAcid. **Throws:**: `MissingAATypeException` - if type is null.: `InvalidIDException` - if residueID is null or does not have at least one non-whitespace character.

---


### addNewHeterogen

```
public Heterogen addNewHeterogen(String name,
                                 String residueID)
                          throws MissingHetNameException,
                                 InvalidIDException
```

:   Creates a new Heterogen with the name and Residue ID given as
    arguments, adds the Heterogen to the Chain's collection of
    Heterogens, and returns a reference to the new Heterogen.

---


### addNewWater

```
public Water addNewWater(String residueID)
                  throws InvalidIDException
```

:   Creates a new Water with the Residue ID given as an argument,
    adds the Water to the Chain's collection of Waters, and returns
    a reference to the new Water.

---


### addNewLoop

```
public Loop addNewLoop(String loopID,
                       String startResidueID,
                       String endResidueID)
                throws InvalidRegionException,
                       InvalidIDException
```

:   Creates a new Loop with the Loop ID given as an argument, adds the
    Loop to the Chain's collection of Loops, and returns a reference
    to the new Loop.

---


### addNewHelix

```
public Helix addNewHelix(String helixID,
                         String serialNo,
                         String startResidueID,
                         String endResidueID,
                         HelixEnum type)
                  throws InvalidRegionException,
                         InvalidIDException
```

:   Creates a new Helix with the Helix ID given as an argument, adds
    the Helix to the Chain's collection of Helices, and returns a
    reference to the new Helix.

---


### addNewBetaStrand

```
public BetaStrand addNewBetaStrand(String betaStrandID,
                                   String betaStrandNumber,
                                   String sheetID,
                                   String startResidueID,
                                   String endResidueID,
                                   int sense,
                                   int strandsInSheet)
                            throws InvalidRegionException,
                                   InvalidIDException
```

:   Creates a new BetaStrand with the BetaStrand ID given as an
    argument, adds the BetaStrand to the Chain's collection of
    BetaStrands, and returns a reference to the new BetaStrand.

---


### sortBetaStrands

```
public void sortBetaStrands()
```

:   After all BetaStrand objects have been added to the Chain, this method can
    be called to sort the linked hash map (the linked list part does have order)
    according to the starting residue for each beta-strand.

---


### getChainID

```
public String getChainID()
```

:   Returns the chainID, which is usually a single letter.
    The String returned cannot be null or empty, because the
    constructor checks that this read-only attribute has at least
    one non-whitespace character.

    :   **Returns:**: The ID of the Chain.

---


### getModelID

```
public String getModelID()
```

:   Returns the modelID of the Model this Chain belongs to.

    :   **Returns:**: The ID of the Model.

---


### getStructureID

```
public String getStructureID()
```

:   Returns the structureID of the Structure this Chain belongs to.

    :   **Returns:**: The ID of the Structure.

---


### getResidue

```
public Residue getResidue(String residueID)
```

:   Returns the Residue with the residueID given as an argument.

    :   **Parameters:**: `residueID` - the unique ID for the desired Residue. **Returns:**: The requested Residue (or null if not found).

---


### getAminoAcid

```
public AminoAcid getAminoAcid(String residueID)
```

:   Returns the AminoAcid with the residueID given as an argument.

    :   **Parameters:**: `residueID` - the unique ID for the desired AminoAcid. **Returns:**: The requested AminoAcid (or null if not found).

---


### getHeterogen

```
public Heterogen getHeterogen(String residueID)
```

:   Returns the Heterogen with the residueID given as an argument.

    :   **Parameters:**: `residueID` - the unique ID for the desired Heterogen. **Returns:**: The requested Heterogen (or null if not found).

---


### getWater

```
public Water getWater(String residueID)
```

:   Returns the Water with the residueID given as an argument.

    :   **Parameters:**: `residueID` - the unique ID for the desired Water. **Returns:**: The requested Water (or null if not found).

---


### getHelix

```
public Helix getHelix(String helixID)
```

:   Returns the Helix with the helixID given as an argument.

    :   **Parameters:**: `helixID` - the unique ID for the desired Helix. **Returns:**: The requested Helix (or null if not found).

---


### getBetaStrand

```
public BetaStrand getBetaStrand(String betaStrandID)
```

:   Returns the BetaStrand with the betaStrandID given as an argument.

    :   **Parameters:**: `betaStrandID` - the unique ID for the desired BetaStrand. **Returns:**: The requested BetaStrand (or null if not found).

---


### iteratorAminoAcids

```
public Iterator<AminoAcid> iteratorAminoAcids()
```

:   Returns an Iterator for the AminoAcids held by this Chain.

---


### iteratorHeterogens

```
public Iterator<Heterogen> iteratorHeterogens()
```

:   Returns an Iterator for the Heterogens held by this Chain.

---


### iteratorWaters

```
public Iterator<Water> iteratorWaters()
```

:   Returns an Iterator for the Water molecules held by this Chain.

---


### iteratorLoops

```
public Iterator<Loop> iteratorLoops()
```

:   Returns an Iterator for the Loops held by this Chain.

---


### iteratorHelices

```
public Iterator<Helix> iteratorHelices()
```

:   Returns an Iterator for the Helices held by this Chain.
      
      
    The order of iteration is the same as the order in which Helices
    were added to the Chain. In the rare case where a Helix was
    replaced (by adding a Helix with the same helixID), the
    replacement would not change the iteration order in any way.

    :   **Returns:**: An Iterator for the Helices held by this Chain.

---


### iteratorBetaStrands

```
public Iterator<BetaStrand> iteratorBetaStrands()
```

:   Returns an Iterator for the BetaStrands held by this Chain.
      
      
    The order of iteration is the same as the order in which
    BetaStrands were added to the Chain. In the rare case where a
    BetaStrand was replaced (by adding a BetaStrands with the same
    betaStrandID), the replacement would not change the iteration
    order in any way.

    :   **Returns:**: An Iterator for the BetaStrands held by this Chain.

---


### numberOfAminoAcids

```
public int numberOfAminoAcids()
```

:   Returns the number of AminoAcids held by this Chain.

    :   **Returns:**: The total number of AminoAcids.

---


### numberOfHeterogens

```
public int numberOfHeterogens()
```

:   Returns the number of Heterogens held by this Chain.

    :   **Returns:**: The total number of Heterogens.

---


### numberOfWaters

```
public int numberOfWaters()
```

:   Returns the number of Waters held by this Chain.

    :   **Returns:**: The total number of Waters.

---


### numberOfLoops

```
public int numberOfLoops()
```

:   Returns the number of Loops held by this Chain.

    :   **Returns:**: The total number of Loops.

---


### numberOfHelices

```
public int numberOfHelices()
```

:   Returns the number of Helices held by this Chain.

    :   **Returns:**: The total number of Helices.

---


### numberOfBetaStrands

```
public int numberOfBetaStrands()
```

:   Returns the number of BetaStrands held by this Chain.

    :   **Returns:**: The total number of BetaStrands.

---


### processID

```
public String processID(String id,
                        String typeOfID)
                 throws InvalidIDException
```

:   Returns the ID after trimming any leading or trailing whitespace.
    An ID must have at least one non-whitespace character.

    :   **Specified by:**: `processID` in interface `IDTest`
    :   **Parameters:**: `id` - ID to process.: `typeOfID` - type of ID (for possible use in error message). **Returns:**: The trimmed ID. **Throws:**: `InvalidIDException` - if the trimmed ID does not have at least one character.

---


### toString

```
public String toString()
```

:   Returns the chainID, which is usually a single letter.
    The String returned cannot be null or empty, because the
    constructor checks that this read-only attribute has at least
    one non-whitespace character.

    :   **Overrides:**: `toString` in class `Object`
    :   **Returns:**: The Chain ID as a String.


---


|  |  |  |
| --- | --- | --- |
| **Overview** \| **Package** \| **Class** \| **Use** \| **Tree** \| **Deprecated** \| **Index** \| **Help** | |  |
| **PREV CLASS**   **NEXT CLASS** | **FRAMES**    **NO FRAMES**     **All Classes** |  |
| SUMMARY: NESTED \| FIELD \| CONSTR \| METHOD | DETAIL: FIELD \| CONSTR \| METHOD |  |


---

# *Copyright © 2007-2008*
